# Discovery and characterization of a novel *CCND1*/*MRCK* gene fusion in mantle cell lymphoma

**DOI:** 10.1186/s13045-016-0260-7

**Published:** 2016-03-29

**Authors:** Chioniso Patience Masamha, Todd R. Albrecht, Eric J. Wagner

**Affiliations:** Department of Pharmaceutical Sciences, College of Pharmacy and Health Sciences, Butler University, 4600 Sunset Avenue, Indianapolis, IN 46208 USA; Department of Biochemistry and Molecular Biology, University of Texas Medical Branch at Galveston, 301 University Boulevard, Galveston, TX 77555 USA

**Keywords:** Mantle cell lymphoma, Gene fusion, Cyclin D1, miRNA, Alternative polyadenylation, 3′UTR

## Abstract

The t(11;14) translocation resulting in constitutive cyclin D1 expression is an early event in mantle cell lymphoma (MCL) transformation. Patients with a highly proliferative phenotype produce cyclin D1 transcripts with truncated 3′UTRs that evade miRNA regulation. Here, we report the recurrence of a novel gene fusion in MCL cell lines and MCL patient isolates that consists of the full protein coding region of cyclin D1 (*CCND1*) and a 3′UTR consisting of sequences from both the *CCND1* 3′UTR and myotonic dystrophy kinase-related Cdc42-binding kinase's (*MRCK*) intron one. The resulting *CCND1/MRCK* mRNA is resistant to *CCND1*-targeted miRNA regulation, and targeting the *MRCK* region of the chimeric 3′UTR with siRNA results in decreased *CCND1* levels.

**Electronic supplementary material:**

The online version of this article (doi:10.1186/s13045-016-0260-7) contains supplementary material, which is available to authorized users.

## Findings

Mantle cell lymphoma (MCL) is considered incurable upon relapse [1]. The hallmark of MCL is the t(11;14)(q13;q32) translocation [2] which results in the constitutive expression of cyclin D1 protein despite the short half-life (~30 min) of its cyclin D1 (*CCND1*) transcript [3]. The *CCND1* mRNA has a long 3′UTR (~3.1 Kb) that contains numerous destabilizing elements [4, 5]. MCL patients with highly proliferative tumors express *CCND1* transcripts with truncated 3′UTRs correlating with reduced survival [6]. In some MCL patients, 3′UTR shortening is due to single nucleotide polymorphisms (SNPs) that result in the generation of an optimal proximal polyadenylation signal (pPAS) [6]. In other cases, no such mutations have been observed, suggesting that alterations in the activity of the cleavage and polyadenylation machinery is responsible. This process, termed alternative polyadenylation (APA) [7], has been the focus of much attention as cancer cells have shown a global tendency to utilize polyadenylation sites proximal to the stop codon to reduce the activity of microRNAs [8]. Aside from SNPs and APA, genomic deletions that result in shortened 3′UTRs have not been well-explored and are the focus of this study [6].

**Fig. 1:**
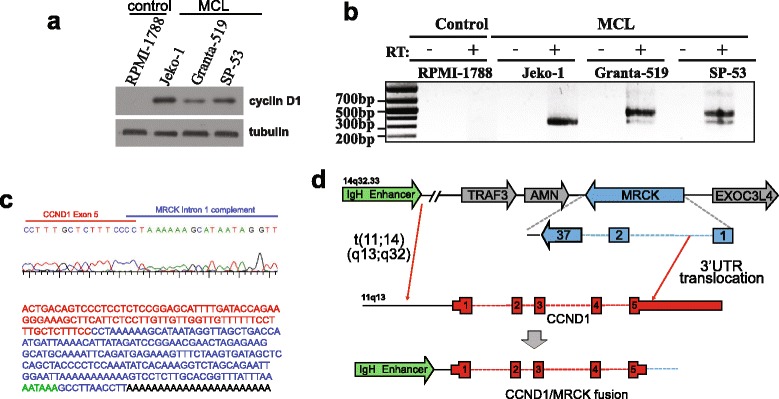
Identification of the novel *CCND1*/*MRCK* fusion gene in mantle cell lymphoma. **a** Western blot analysis confirms cyclin D1 protein expression in three MCL cell lines but not in the normal B cell line RPMI 1788. **b** PCR products derived from 3′RACE of RNA isolated from RPMI-1788, Jeko-1, Granta-519, and SP-53 cell lines. **c** A partial sequence shows the junction between the *CCND1* and *MRCK* sequences after blunt-end cloning and sequencing. *CCND1* sequence is in *red*, *MRCK* is in *blue*, the canonical poly(A) signal is in *green*, and the poly(A) tail is in *black*. **d** Schematic representation of the t(11;14) translocation as well as the second translocation of *CCND1* and *MRCK* to create the fusion gene

We confirmed aberrantly high cyclin D1 protein expression in all three MCL cell lines compared to a control B lymphocyte cell line, RPMI-1788 (Fig. 1a). To determine the state of the 3′UTR, we mapped the 3′end of *CCND1* mRNA. We observed 3′rapid amplification of cDNA end (3′RACE) products in all the MCL lines that would indicate that 3′UTR shortening has occurred (Fig. 1b). Jeko-1 utilizes a non-consensus and unmutated pPAS (AATAAT) (Additional file 1). Interestingly, mutations in this same genomic region creating a consensus pPAS (AATAAA) have been observed in 3/15 MCL patients [6]. Hence, in Jeko-1, APA is the likely cause of *CCND1* 3′UTR shortening to allow for the use of a non-optimal pPAS.

Unexpectedly, in Granta-519 and SP-53, the 3′RACE products were slightly larger than that observed from Jeko-1. Sequencing revealed that, in both cases, the 3′UTR of *CCND1* is fused to the reverse complement of intronic sequences present in the myotonic dystrophy kinase-related Cdc42-binding kinase (*MRCK*) gene (Fig. 1c). Placement of this genomic region within the *CCND1* 3′UTR results in the use of a consensus polyadenylation signal (PAS) from *MRCK*, which triggers the subsequent addition of the poly(A) tail, creating a chimeric 3′UTR. The observed *CCND1*/*MRCK* fusion gene likely is formed by a second translocation event between chromosomes 11 and 14, which positions the full open reading frame of *CCND1* and a truncated 3′UTR within intron one of *MRCK* (Fig. 1d).

**Fig. 2:**
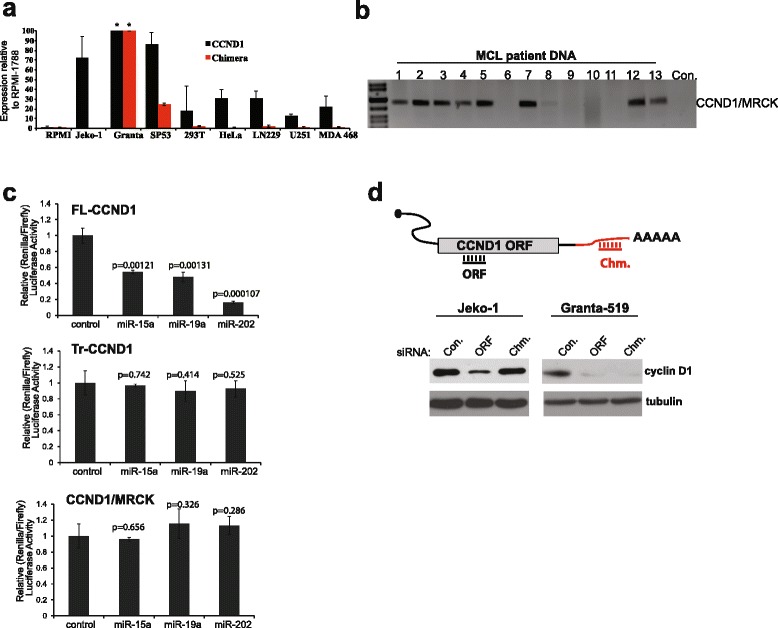
The *CCND1*/*MRCK* fusion is refractory to *CCND1*-targeted miRNA regulation and can be targeted for RNAi. **a** Quantitative real-time PCR (qRT-PCR) on mRNA extracted from different cell lines showing the expression levels of total *CCND1* and the *CCND1/MRCK* chimera in each cell relative to levels in RPMI 1788. The * denotes expression levels of 374 and 347 for *CCND1* and the chimeric mRNA, respectively. **b** Detection of *CCND1/MRCK* in MCL patient-derived DNA using PCR. **c** Dual luciferase reporter assay of HeLa cells after transfection with different miRNA mimics (48 h-post transfection) and plasmids (24 h-post transfection) containing the full-length 3′UTR of *CCND1* (FL-*CCND1*), the Jeko-1 specific truncated *CCND1* 3′UTR (Tr-*CCND1*) and the *CCND1/MRCK* fusion 3′UTR. Shown are representative results of experiments performed in triplicate, with each miRNA compared to control using the *t* test. **d** A schematic showing the location of the sequences targeted by siRNAs with specific siRNAs targeting the protein-coding sequence of *CCND1* (ORF) and the *MRCK* sequence from the *CCND1/MRCK* fusion (Chm). Western blot of cell lysates after nucleofection of Jeko-1 and Granta-519 with specific siRNAs targeting the protein-coding sequence of *CCND1* (ORF) and the *MRCK* sequence from the *CCND1/MRCK* fusion (Chm)

The presence of the *CCND1*/*MRCK* chimeric mRNA was validated using chimera-specific primers (Additional file 1). Quantitative polymerase chain reaction (qRT-PCR) shows that MCL cell lines express at least twice as much *CCND1* mRNA as other cancer cell lines (Fig. 2a). Surprisingly, we were able to detect the *CCND1*/*MRCK* fusion product in 8 out of 13 MCL patient DNA samples (Fig. 2b). These results suggest that this translocation event may be selected for in MCL and functions as a mechanism to shorten the *CCND1* 3′UTR.

StarBase analysis [9] identified 86 miRNAs that can potentially interact with the *CCND1* 3′UTR (Additional file 2). To determine the impact of the *CCND1*/*MRCK* fusion on miRNA regulation of *CCND1*, we generated a reporter plasmid with the chimeric 3′UTR placed downstream of luciferase. We also made constructs containing either the full-length (FL-*CCND1*) or the truncated *CCND1* 3′UTR from Jeko-1 (Tr-*CCND1*) and co-transfected them with three miRNA mimics known to repress *CCND1*. The FL-*CCND1* reporter was downregulated in response to each mimic tested (Fig. 2c). However, all three mimics had no significant effect on either the Tr-*CCND1* or *CCND1*/*MRCK* reporters. This observation parallels reports in glioblastoma where a recurrent *FGFR3*/*TACC3* causes the truncation of the *FGFR3* 3′UTR, resulting in elimination of miRNA binding sites [10]. Intriguingly, transfection of siRNA-targeting of the *MRCK* component of the chimeric 3′UTR causes reduced cyclin D1 protein expression as effectively as siRNA targeting the *CCND1* sequences (Fig. 2d).

These results provide an enhanced understanding of the strength of selection for *CCND1* 3′UTR shortening that occurs in MCL. In addition, the novel *CCND1*/*MRCK* gene fusion identified is a potential new diagnostic and/or therapeutic target for MCL.

## Methods

### Cell lines and cell culture

All of the MCL cell lines and the normal B-lymphocyte line, RPMI 1788, were cultured in RPMI media with 20% fetal bovine serum (FBS) and 5% penicillin/streptomycin. All three MCL cell lines: Jeko-1, Granta-519, and SP-53 contain the t(11;14)(q13;q32) translocation [11]. The remainder of the cell lines used in this study: HeLa, 293T, LN229, U251, and MDA MB 468 were cultured in DMEM (with 10% FBS and 5% Pen/Step).

### Patient samples and sample preparation

All the samples from MCL patients were obtained as deidentified DNA from the Indiana Biobank, a biorepository run by Indiana University. These patients had all been diagnosed with MCL with ICD-9 codes from 200.40 to 200.48. All specimens were collected by Indiana Biobank from patients after informed consent, as approved by the Indiana University Institutional Review Board. PCR was done using Phusion DNA Polymerase (New England Biolabs) per the manufacturer’s protocol. HF Phusion buffer was used together with MgCl2. Primers were designed to provide the largest DNA PCR product possible, and were validated to work with genomic DNA. The primer sequences used were CCND1 forward 5´TCCGGAGCATTTTGATACCAG and MRCK reverse 5´TCCAATTCTGCTAGACCTTTGTGATA.

### mRNA extraction, 3’RACE, PCR and qRT-PCR

Total mRNA was extracted using TRIzol reagent (Life Technologies). After DNase (Promega) treatment, cDNA synthesis was carried out using M-MLV Reverse Transcriptase (Life Technologies). For 3´RACE, oligo(dT25)T7 primer was used in cDNA synthesis, and the first round of PCR was performed using the CCND1 primer 5´ TGGTGAACAAGCTCAAGTGG and oligo(dT25)T7. The second round of PCR was performed using a nested CCND1 primer 5´TGGCATTTTGGAGAGGAAGTG and a T7 primer. All PCR was performed using pfu polymerase. The resulting PCR product was cloned using Zero Blunt TOPO PCR Cloning Kit for Sequencing (Life Technologies) and sequenced (Lonestar Labs, TX). The qRT-PCR protocol used, as well as the amplicons used to measure 7SK and CCND1, were previously described [8]. The primers used to detect CCND1-MRCK were the CCND1 forward primer 5´ GAGGAGGAAGAGGAGGAGGAGGAGGT and the MRCK reverse primer 5´TCCAATTCTGCTAGACCTTTGTGATA. Primers used to detect the control 18S rRNA were 5´CAGCCACCCGAGATTGAGCA and 5´TAGTAGCGACGGGCGGTGTG.

### Cloning of 3´UTR into psicheck 2 dual luciferase plasmid

Three different CCND1 3´UTR constructs were cloned downstream of the Renilla luciferase gene of psicheck2 (Promega) between Xho1 and Not1. To clone the gene fusion, the CCND1-MRCK sequence: CTCGAGGGGCGCCAGGACGGCGGGCGCCACCGCCACCCGCAGCGAGGGCGGAGCCGGCCCCAGGTGCTCCCCTGACAGTCCCTCCTCTCCGGAGCATTTTGATACCAGAAGGGAAAGCTTCATTCTCCTTGTTGTTGGTTGTTTTTTCCTTTGCTCTTTCCCCTAAAAAAGCATAATAGGTTAGCTGACCAATGATTAAAACATTATAGATCCGGAACGAACTAGAGAAGGCATGCAAAATTCAGATGAGAAAGTTTCTAAGTGATAGCTCCAGCTACCCCTCCAAATATCACAAAGGTCTAGCAGAATTGGAATTAAAAAAAAAAAGTCCTCTTGCACGGTTTATTTAAAATAAAGCCTTAACCTTAGGTGGTGCACCAAGTTGAACCTGACAGTGGAACTGTGTGGGTTTCAAGATCGAGTGATCAGAAAGGAACGGTAAACAAGCTGGGTGCAGTGGCTCACGCCTGTAATCCCAGCACTTTGGGAGGCCGAGGCAGGTGGATCACCCGAGGTCAGGAGTACAAGACCAGCCTGGCCAACACTGTGGCGGCCGC was synthesized by GenScript and ligated into pUC57-Kan. After Xho1 and Not1 Restriction enzyme digestion, the sequence was directly cloned into the psicheck2 plasmid. The Jeko-1 specific truncated 3´UTR was cloned from Jeko-1 cDNA using the following primers: forward 5´GGCCCTCGAGGGGCGCCAGGCAGGCGGGCGC and reverse 5´GGCCGCGGCCGCTGCCTAGAACCCCACTACAGCTGTGC. Full length CCND1 3´UTR was cloned from the CCND1-pLightSwitch 3´UTR plasmid (S813994) from SwitchGear Genomics. The primers used were: forward 5´ GGCCGTCGACGGGCGCCAGGCAGGCGGGCGC and reverse 5´ GGCCGCGGCCGC CGTCTTTTTGTCTTCTGCTGGA.

### RNAi and luciferase assays

Either control siRNA [8], CCND1-specific siRNA (SASI Hs 01-00213909 from Sigma), or MRCK target- specific siRNA, (5´ GAUCCGGAACGAACUAGAGTT) were electroporated into MCL cells using the Nucleofactor Kit R (Amaxa), following the manufacturer’s protocol (program A-023 on Nucleofactor II device). Warm cell culture media (500ul) was added after electroporation, and the cells were replated and harvested for Western blot 72 hours later. To conduct luciferase assays, HeLa cells were plated (4.5x104 per well) in a 24 well plate. After 24 hours, cells were transfected with 100ng of microRNA mimics using Lipofectamine 2000 (Life technologies). The MISSION microRNA mimics used were: miR-15a, miR-19a, miR-202, and the miRNA mimic negative control #2 (Sigma). After 24 hours, the cells were then transfected with 50ng of psi-check-2 dual luciferase plasmid hours. The cells were lysed one day after plasmid transfection, and luciferase assays were performed using the Dual-Luciferase Reporter Assay System (Promega).

### Western blot

Cells were lysed using RIPA buffer and 20ug of each protein sample was resolved on a 12% SDS-PAGE gel. After transfer to a PVDF membrane, blocking was done for 1hr in 5% non-fat milk resuspended in PBS (+0.001 % Tween 20). The membranes were probed with the monoclonal cyclin D1 antibody (DCS6 from Cell Signaling) and polyclonal alpha tubulin antibody (Abcam). After probing with HRP-conjugated secondary antibody, proteins were detected by luminol.

### miRNA identification

We used predicted CCND1-miRNA interactions using starBase software, which consolidates TargetScan, PicTar, PITA, Miranda and RNA22 miRNA prediction softwares, and overlaps the data with CLIP-Sequencing (CLIP-Seq.) data. We used a high stringency cutoff, where only miRNAs supported by >/=3 CLIP-Seq. experiments were selected, to reduce false positives [9].

## Additional files

Additional file 1:
**a** Sequence of PCR product derived from Jeko-1 with the annotated PAS (yellow highlight) and unmutated PAS (green highlight) showing the position of the poly(A) tail. **b** PCR analysis using chimera specific *CCND1/MRCK* (chimera) of total RNA isolated from Jeko-1, Granta-519, and SP-53 cells. Results of triplicate biological replicates are shown. (EPS 1874 kb) Additional file 2: Table 1 of *CCND1* miRNA interactions. (XLSX 16 kb)

## Abbreviations

3′RACE: 3′rapid amplification of cDNA ends
CCND1: cyclin D1
Chm: chimera
FGFR: fibroblast growth factor receptor
IgH: immunoglobulin heavy chain
MCL: mantle cell lymphoma
MRCK: myotonic dystrophy kinase-related Cdc42-binding kinase
PAS: polyadenylation signal
pPAS: proximal polyadenylation signal
qRT-PCR: quantitative real-time polymerase chain reaction
RNAi: RNA interference
SNPs: single nucleotide polymorphisms
TACC3: transforming acidic coiled-coil containing protein 3
